# Hyperspectral Imaging to Study Dynamic Skin Perfusion after Injection of Articaine-4% with and without Epinephrine—Clinical Implications on Local Vasoconstriction

**DOI:** 10.3390/jcm10153411

**Published:** 2021-07-31

**Authors:** Daniel G. E. Thiem, Lukas Hans, Sebastian Blatt, Paul Römer, Diana Heimes, Bilal Al-Nawas, Peer W. Kämmerer

**Affiliations:** 1 Department of Oral and Maxillofacial Surgery, University Medical Centre Mainz, 55131 Mainz, Germany; lukashans@gmx.de (L.H.); sebastian.blatt@unimedizin-mainz.de (S.B.); paul.roemer@unimedizin-mainz.de (P.R.); diana.heimes@unimedizin-mainz.de (D.H.); bilal.al-nawas@unimedizin-mainz.de (B.A.-N.); peer.kaemmerer@unimedizin-mainz.de (P.W.K.); 2 Department of Oral and Maxillofacial Surgery, School of Dentistry, Kyung Hee University, Seoul 05278, Korea

**Keywords:** hyperspectral imaging, vasoconstriction, dosage form, injection-to-cut-time, ischaemia, hyperaemia, skin perfusion, non-invasive, contactless, same effect

## Abstract

This study aimed to investigate the dynamic skin perfusion via hyperspectral imaging (HSI) after application of Articaine-4% ± epinephrine as well as epinephrine only. After the subcutaneous injection of (A100) Articaine-4% with epinephrine 1:100,000, (A200) Articaine-4% with epinephrine 1:200,000, (Aw/o) Articaine-4% without epinephrine, and (EPI200) epinephrine 1:200,000, into the flexor side of the forearm in a split-arm design, dynamic skin perfusion measurement was performed over 120 min by determining tissue oxygen saturation (StO_2_) using HSI. After injection, all groups experienced a reactive hyperaemia. With A200, it took about three min for StO_2_ to drop below baseline. For Aw/o and EPI200, perfusion reduction when compared to baseline was seen at 30 min with vasoconstriction >120 min. A100 caused vasodilation with hyperaemia >60 min. After three minutes, the perfusion pattern differed significantly (*p* < 0.001) between all groups except Aw/o and EPI200. The vasoactive effect of epinephrine-containing local anaesthetics can be visualised and dynamically quantified via StO2 using HSI. Aw/o + epinephrine 1:100,000 and 1:200,000 leads to perfusion reduction and tissue ischaemia after 30 min, which lasts over 120 min with no significant difference between both formulations. When using Aw/o containing epinephrine in terms of haemostasis for surgical procedures, a prolonged waiting time before incision of 30 or more min can be recommended.

## 1. Introduction

The use of local anaesthetics (LA) containing vasoconstrictors is common practice in dentistry and oral and maxillofacial surgery [1,2]. However, other surgical disciplines such as dermatosurgery or hand surgery also appreciate the advantages of vasoconstrictive additives i.e., increase of local anaesthetic duration, providing haemostasis for surgical procedures and to reduce systemic local anaesthetic blood levels [3,4]. Once developed to prolong anaesthesia, it simultaneously provides ischaemia in the surgical field [5]. Simultaneously, prolonged vasoconstriction can cause tissue infarction [6,7]. Particularly at risk and therefore even declared contraindicated for the use of epinephrine-containing LA are fingers, ears, nose, or penis [8]. However, studies have consistently failed to support this theory, which has led to the development of surgical procedures such as the “Wide-Awake-Local-Anaesthesia-No-Torniquet” (WALANT) methods based on epinephrine-containing LA [9]. Epinephrine has long been the community standard for local vasoconstriction, but it has some limitations due to potential cardiovascular and local toxic effects [10]. Since its complications are mainly dose-dependent, care should be taken when choosing vasoconstrictor-containing local anaesthetics to keep the epinephrine concentration as low as possible [1,11]. This is particularly important when alpha 1-adrenoreceptor mediated vasoconstriction is used to intentionally reduce blood flow to the surgical field. Moore et al. were able to show in their randomised, split-mouth, double-blind study that the application of 4% Articaine with epinephrine 1:100,000 resulted in less blood loss and a more bloodless surgical field than with 4% Articaine and epinephrine 1:200,000 [3].With regard to the vasoactive effect and the associated influence on the microcirculation, a subjective evaluation of the ischaemia-related colour change, colour doppler flow imaging, and pulse oximetry was carried out in most available studies [12]. However, there is an ongoing debate especially concerning the time to onset and duration of vasoconstriction with respect to the used drug combinations and dilutions [13]. Hyperspectral imaging (HSI) is a non-contact, non-ionising, and non-invasive technique that provides objective, reproducible, precise information about parameters used for tissue perfusion measurements and wound assessment [14,15,16]. HSI processes the optical properties of a large wavelength range from visual light (380–740 nm) to near infrared (750–1000 nm; NIR), acquiring a 3D dataset (“hypercube”) [16]. The cameras’ system software is trained to detect and measure haemoglobin with its derivatives oxyhaemoglobin (O_2_Hb) and deoxyhaemoglobin (HHb) in order to analyse the cutaneous and subcutaneous (s.c.) oxygenation pattern (StO_2_) [16]. This study is the first to investigate the vasoactive effect of a LA (Articaine-4%) with different concentrations of added epinephrine (1:100,000, 1:200,000, without) as well as plain epinephrine via dynamic visual and quantitative measurements.

## 2. Materials and Methods

### 2.1. Study Cohort

A total of 50 healthy volunteer adults participated in this methodological basic research study. The study included males and females between 18 and 65 years, without known cardiovascular diseases, allergies, or intolerances to the ingredients of the LA solutions used, history of intolerance to LA, or other epinephrine-containing drugs. All patients who did not meet the inclusion criteria were not included.

### 2.2. Test Substances and Subcutaneous Application

**Figure 1:**
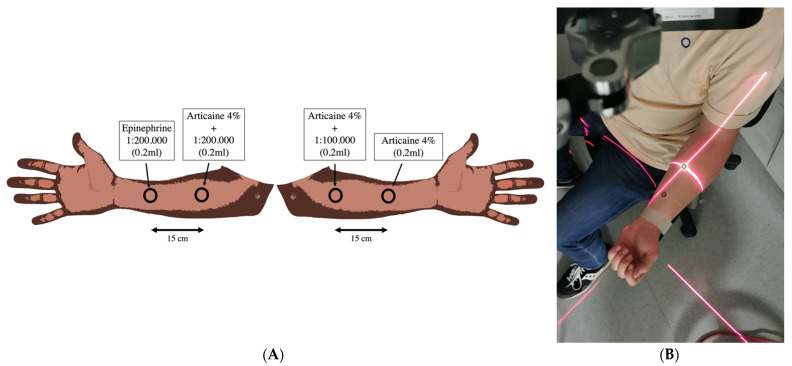
Schematic illustration of the split-arm design with rubber ring and centrally located application site with a minimum distance of 15 cm between the injection sites (**A**). The test set-up with the test person sitting and the distance laser switched on with the focus on the measuring point (**B**).

The experiments were conducted in a laboratory at an environmental temperature of 22 °C, and the participants were seated with their arms supported at heart level. Each participant’s baseline blood pressure was determined before and during the experiment. Each participant received 0.2 mL of (A100) Articaine-4% +epinephrine 1:100,000 (40 mg/mL articaine + 0.012 mg/mL epinephrine hydrochloride), (A200) Articaine-4% +epinephrine 1:200,000 (40 mg/mL articaine and 0.006 mg/mL epinephrine hydrochloride; Ultracain^®^D-S, Sanofi-aventis, Paris, France), (Aw/o) plain Articaine-4% (40 mg/mL articaine; Ultracain^®^D, Sanofi-aventis, Paris, France) and (EPI200) epinephrine hydrochloride 1:200,000 (0.006 mg/mL; Epinephrine (Epinephrinehydrogentartrat) 1:1000, INFECTOPHARM^®^, Heppenheim, Germany). Each drug was applied subcutaneously at a puncture angle of 30° to the skin surface in a “split-arm design” with two substances per forearm and side (15 cm from each other to avoid overlapping). The measuring points were marked with a black rubber ring (6.7 mm inner diameter) (Figure 1A,B).

### 2.3. Hyperspectral Imaging (HSI)

**Figure 2:**
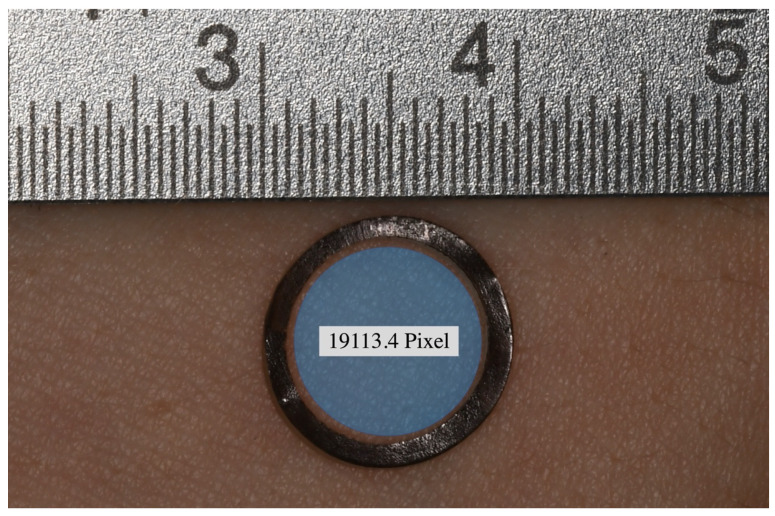
The rubber ring with scale and the highlighted (blue) region of interest (ROI-1) with a circular area of 19,113.4 pixels.

**Figure 3:**
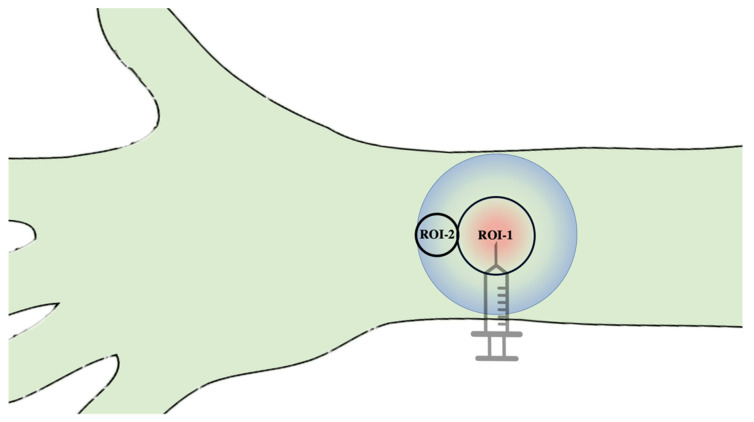
Schematic illustration of the two regions of interest and their positional relationship to each other.

In this study, a novel hyperspectral camera (TIVITA^®^, Diaspective Vision GmbH, Am Salhaff, Germany), specially developed for extracorporeal perfusion diagnostics, was used as described by our group beforehand [17,18]. The camera system is composed of a 120 W halogen illumination source and a radiometrically calibrated 32-bit complementary metal-oxide semiconductor spectrometer, capturing images at a resolution of 480 × 640 pixels. The hyperspectral cube contains 100 spectral bands, from 500 to 1000 nm with a 5 nm sampling interval. Briefly, HSI is based on the assessment of contiguous spectra, molecule-specifically re-emitted, on the basis of the light spectrum of the halogen spotlights initially emitted for examination. After hyperspectral images are recorded, an additional 8 s are needed to compute an RGD (red, green, and blue) true-colour image and four pseudo-colour images, representing the following: tissue-oxygenation-saturation/superficial perfusion (StO_2_ (0–100%)), near-infrared-perfusion index/deep perfusion (NIR as arbitrary units (0–100)) and distribution of haemoglobin (Tissue-Haemoglobin-Index (0–100)) and water (Tissue-Water-Index (0–100)) [19]. The measured oxygen saturation is the percentage of oxygen bound to haemoglobin. This is important for the determination of tissue hypoxia, since the amount of dissolved oxygen in the tissue can be shown by measuring oxygen saturation due to the fact that the oxygen binding curve relates oxygen saturation to a certain amount of dissolved oxygen. If one wants to know how much oxygen is absolutely available, one needs to know the blood flow in addition to the oxygen saturation. Only then, the absolute inflow (blood flow and arterial oxygen saturation) and outflow (blood flow and the capillary–venous oxygen saturation) can be used to determine the amount of oxygen delivered to the tissue. The black rubber rings’ radius was 78 pixels (equivalent to 3.3 mm at 72 dpi), corresponding to the region of interest (ROI-1), which comprised a slightly smaller area of 19,113.4 pixels (33.8 mm^2^; Figure 2). In order to assess the propagation dynamics of the different drug formulations (A100, A200, Aw/o, EPI200), an additional analysis of an adjacent circular area of 2827.4 pixels (5 mm^2^) in 90° position (ROI-2) to the application direction vector through the centre of ROI-1 was performed (Figure 3). Here, no additional acquisition was necessary, since the peripheral measurement area was still in the image area and as such could be quantified separately by means of a separate region of interest. The HSI of each region to be analysed was taken before injection and served as a baseline. Then, the measurements were taken at the specified time points post injection (p.i.) at 30 s, 1, 2, 3, 4, 5, 15, 30, 45, 60, and 120 min. StO_2_ is measured 1 mm below the skin surface, and it is considered a valid surrogate parameter for microcirculatory tissue perfusion.

### 2.4. Statistics

In this feasibility study, the null hypothesis was that the vasoconstrictive effect of epinephrine (with and without LA) does not differ with regard to its concentration. In addition, it was hypothesised that the onset and duration of this effect on perfusion is not influenced by the epinephrine concentration. The parameters to be measured were the dynamic blood flow in superficial layers of the skin that can be objectively and reproducibly quantified and visualised by means of hyperspectral imaging. With an average assumed effect size (dz = 0.6/mean effect size according to Cohen (1988)), an α error = 0.05 and a power = 95%, the necessary sample size was (*n* = 39 (40)) [20]. Therefore, a *n* = 50 per solution was chosen for the present study. Raw datasets were saved in Excel^®^ sheets (Microsoft Corporation, Redmond WA, USA) and subsequently transferred into SPSS-Statistics^®^ (version 27 MacOS X; SPSS Inc., IBM Corporation, Armonk, NY, USA). Data were expressed as mean (m), standard deviation (SD±), minimum, and maximum. Normal distribution was checked using a non-parametric Shapiro–Wilk-test^(+)^, and the results were analysed for statistical significance by the use of analysis of variance (ANOVA^(#)^), unpaired non-parametric Mann–Whitney U-tests^($)^, Wilcoxon Signed Ranks test^(§)^, and Student’s *t*-test^(^*^)^. To investigate whether the means of several dependent samples differ, we performed a one-factor analysis of variance with repeated measures. In the absence of sphericity (Mauchly W-test <0.05) and an epsilon correction factor Greenhouse–Geisser test of >0.75, the epsilon correction factor and significance according to Huynh–Feldt (HF) was chosen (^§^*). The partial Eta square (η_p_^2^) shows how much percent of the variation of StO_2_ can be explained by the time of measurement. *p*-values of ≤0.05 were termed significant. Line charts were used for illustration purposes.

## 3. Results

### 3.1. Central^ROI−1^ and Peripheral^roi−2^ Perfusion Pattern (Sto_2_) over Time

#### 3.1.1. Baseline

*Central^ROI−1^*

Before injection, baseline tissue oxygen saturation (StO_2_) did not differ significantly between substance groups/measurement points (mean: 40.7315% ± 5.62) (*p* = 0.827^(#)^).

*Peripheral^ROI−2^*

There was no significant difference between all groups at baseline (mean: 41.36 ± 5.4) (*p* = 0.220^(#)^).

*Central^ROI−1^* vs. *Peripheral^ROI−2^*

**Figure 4:**
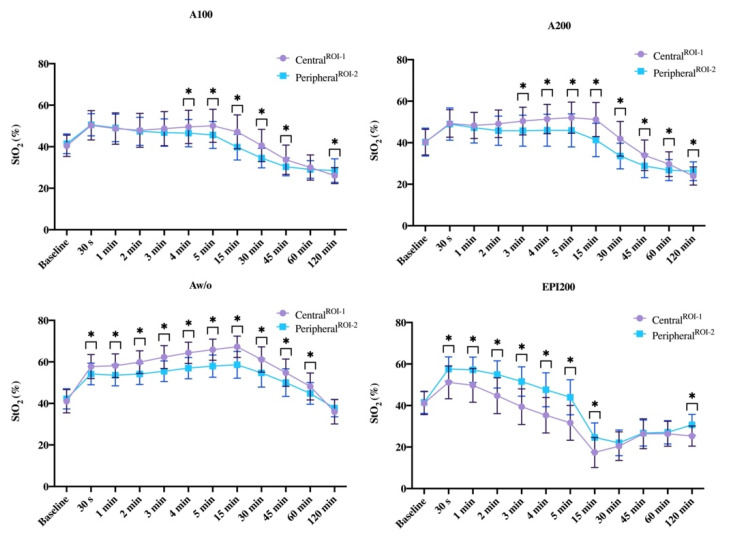
Line graphs show the group-specific course of StO_2_ at the measurement time points comparing ROI-1 and ROI-2. Asterixis mark the statistically significant difference of StO_2_ at the respective measurement time point. The values are shown as means with standard deviation (±SD).

Before injection, there was no difference in cutaneous perfusion between ROI-1 and ROI-2 (Figure 4, Table S1). 

#### 3.1.2. 30. s p.i.

*Central^ROI^* ^−1^

Thirty seconds p.i., blood flow was significantly increased with Aw/o compared to A100 and A200 (each *p* ≤ 0.001^($)^) and EPI200 (*p* ≤ 0.001).

*Peripheral^ROI−2^*

**Figure 5:**
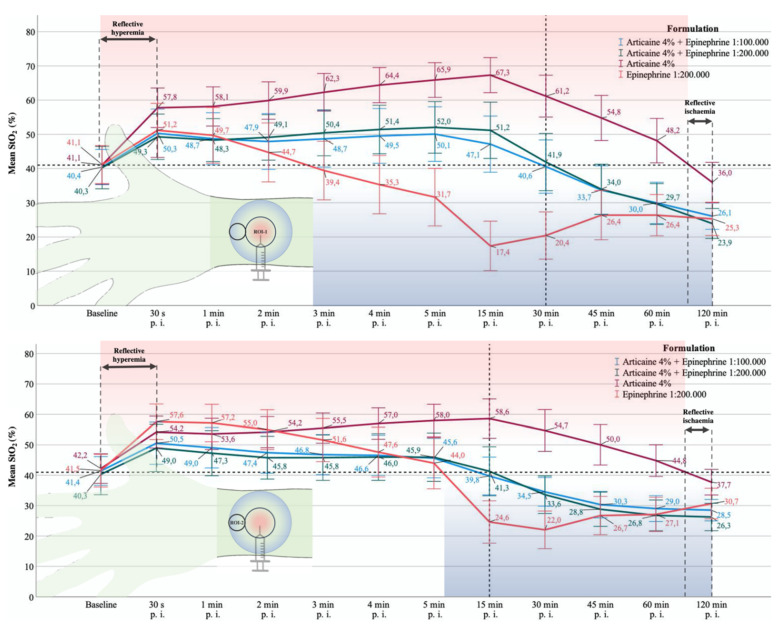
Development over time (X-axis) of cutaneous blood flow by means of StO_2_ (Y-axis) for ROI-1 (**upper**) and ROI-2 (**lower**). The values are shown as mean value with standard deviation (±SD).

StO_2_ was significantly different between Aw/o and A100 and A200 (each *p* < 0.001^($)^), and EPI200 (*p* = 0.011^($)^), whereby values revealed significantly increased with EPI200 when compared to A100 and A200 (each *p* < 0.001^($)^) (Figure 5).

*Central^ROI−1^* vs. *Peripheral^ROI−2^*

StO_2_ was higher in ROI-2 with Aw/o (*p* = 0.003^($)^) and EPI200 (*p* < 0.001^($)^) compared to ROI-1. No difference occurred for A100 and A200 (Figure 4, Table S2).

#### 3.1.3. One and Two Minutes p.i.

*Central^ROI−1^*

After 1 and 2 min, StO_2_ was significantly increased with plain Articaine-4% versus A100 (*p* ≤ 0.001^($)^) and A200 (*p* ≤ 0.001^(#)^) and EPI200 (*p* ≤ 0.001^($)^). No significant difference was found between A100 and A200 and between EPI200 and A100 (Figure 5).

*Peripheral^ROI−2^*

StO_2_ was significantly increased with Aw/o when compared to A100 and A200 (each *p* < 0.001^($)^), which revealed a significant lower StO_2_ (*p* < 0.001^($)^) when compared to EPI200 (Figure 5, Table S3).

*Central^ROI−1^* vs. *Peripheral^ROI−2^*

After 1 and 2 min, StO_2_ was significantly higher peripherally than centrally with Aw/o and EPI200 (*p* < 0.001^($)^). No difference occurred for A100 and A200.

#### 3.1.4. Three, Four, and Five Minutes p.i.

*Central^ROI−1^*

After 3, 4, and 5 min, StO_2_ decreased significantly with EPI200 in comparison to Aw/o, A100, and A200 (each *p* ≤ 0.001^($)^). Simultaneously, StO_2_ significantly increased with Aw/o compared to A100 and A200 (each *p* ≤ 0.001^($)^); Table S4). There was no significant difference for A100 and A200. With EPI200, StO_2_ dropped below baseline between 2 and 3 min p.i. (Figure 5).

*Peripheral^ROI−2^*

Three minutes p.i., StO_2_ differed significantly (*p* < 0.001^($)^) between the agents in the same proportion as centrally (ROI-1). In contrast, StO_2_ after 4 min was not significantly different between Aw/o + 1:100,000 and A200 and EPI200, but it was significantly higher (*p* < 0.001) with Aw/o compared to the other groups, which persisted until five minutes after injection (Figure 5).

*Central^ROI−1^* vs. *Peripheral^ROI−2^*

StO_2_ differed significantly between ROI-1 and ROI-2 for all agents equally (*p* < 0.001^($)^) except A100. Centrally, StO_2_ was higher for the vasoconstrictor-containing LA formulations and lower for EPI200 (Figure 4; Table S4). After 4 and 5 min, StO_2_ differed significantly with A100 between ROI-1 and 2 (*p* = 0.029^($)^; *p* < 0.001^($)^) (Figure 4; Table S4).

#### 3.1.5. 15 min p.i.

*Central^ROI−1^*

StO_2_ was significantly different when comparing all test substances, with the highest values for Aw/o, followed by A100 and A200 and EPI200 (Figure 5).

*Peripheral^ROI−2^*

StO_2_ differed significantly (*p* < 0.001^($)^; *p* = 0.041^($)^) between all groups (Figure 5). In contrast to ROI-1, StO_2_ dropped to baseline after 15 min *p.i.* with A100 and A200.

*Central^ROI−1^* vs. *Peripheral^ROI−2^*

Local blood flow (StO_2_) remained significantly higher in ROI-1 for Aw/o and A100 and A200 (*p* < 0.001^($)^) and significantly lower for epinephrine (*p* < 0.001^($)^), compared to ROI-2 (Figure 4; Table S5).

#### 3.1.6. 30 min p.i.

*Central^ROI−1^*

StO_2_ dropped to baseline level with A100 and A200 (Figure 5). StO_2_ remained significantly lower with A100 and A200 when compared to Aw/o (*p* ≤ 0.001^($)^), and significantly higher when compared to EPI200 (*p* ≤ 0.001^($)^).

*Peripheral^ROI−2^*

In ROI-2, StO_2_ was similar to ROI-1 with no difference between the vasoconstrictor-containing LA-solutions A100 and A200 (*p* = 0.238^($)^). In all other groups, the blood flow was significantly (each: *p* < 0.001^($)^) different. In summary, between 15 and 30 min after injection, there was a decrease in the previously increasing blood flow with Aw/o and a further decrease with A100 and A200, as well as with EPI200 (Figure 4, Table S6).

*Central^ROI−1^* vs. *Peripheral^ROI−2^*

Thirty min after injection, StO_2_ was still significantly higher in ROI-1 with Aw/o, as well as with A100 and A200 when compared to ROI-2 (*p* < 0.001^($)^), whereby the latter two peripherally dropped below baseline into the ischemic zone. Although not significant, there was an increase in StO_2_ in ROI-1 with EPI200 (Figure 4).

#### 3.1.7. 45 min after Injection

*Central^ROI−1^*

After 45 min, a clear drop of StO_2_ below baseline in terms of local ischaemia was evident with A100 and A200 (Figure 2); however, a clear difference to Aw/o (lower) and EPI200 (higher) was still significant (each *p* ≤ 0.001^($)^). While a pronounced increase in StO_2_ in the sense of hyperaemia was still evident with Aw/o, StO_2_ was significantly lower with EPI200 (*p* ≤ 0.001^($)^).

*Peripheral^ROI−2^*

StO_2_ was significantly higher with Aw/o compared to EPI200 and A100 and A200 (each: *p* < 0.001^($)^). Values were also different between A100 and A200 (*p* = 0.046^($)^).

*Central^ROI−1^* vs. *Peripheral^ROI−2^*

Perfusion differed significantly between ROI-1 and ROI-2 with Aw/o, A100, and A200 (*p* < 0.001^($)^; *p* = 0.003^($)^; *p* < 0.001^($)^), whereas EPI200 revealed almost the same (Figure 4; Table S7).

#### 3.1.8. 60 min after Injection

*Central^ROI−1^*

There were significant differences in StO_2_ between all groups except A100 and A200 (*p* = 0.91^($)^).

*Peripheral^ROI−2^*

StO_2_ was significantly different (*p* < 0.01^($)^) between all substances except for EPI200 and A200 (Figure 4).

*Central^ROI−1^* vs. *Peripheral^ROI−2^*

StO_2_ was similar in ROI-1 and ROI-2 A100 and Epi200, whereas it differed significantly for Aw/o (*p* = 0.009^($)^) and A200 (*p* = 0.002^($)^) comparing ROI-1 and ROI-2 (Figure 4; Table S8).

#### 3.1.9. 120 min after Injection

*Central^ROI−1^*

It revealed a significant difference in StO_2_ between A100 and A200 (*p* = 0.006). There was also a significant difference in StO_2_ between Aw/o and all other groups (*p* ≤ 0.001^($)^).

*Peripheral^ROI−2^*

StO_2_ dropped below baseline with Aw/o, which differed significantly from A100 and A200, and from EPI200 (*p* < 0.001^($)^) but not from baseline. In contrast, StO_2_ was significantly higher with EPI200 compared to A100 (*p* = 0.005^($)^) and A200 (*p* < 0.001^($)^).

*Central^ROI−1^* vs. *Peripheral^ROI−2^*

Except for Aw/o, StO_2_ was significantly different (*p* < 0.001^($)^) between ROI-1 and ROI-2 for all agents (Figure 4; Table S9).

#### 3.1.10. Difference to Baseline StO2

*Central^ROI−1^*

**Figure 6:**
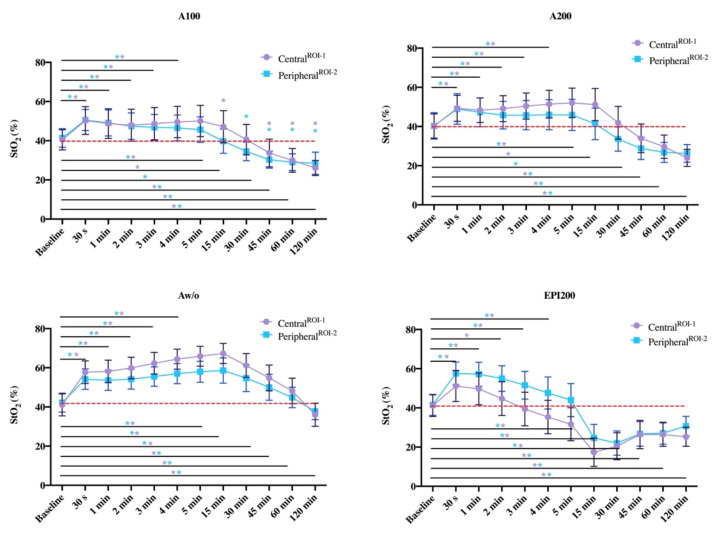
Line graphs show the difference of each measurement time point from baseline separately for each group when ROI-1 and ROI-2 are plotted simultaneously. This is indicated by the asterisks inserted in the colour of the line graphs. The values are shown as means with standard deviation (±SD).

Overall, for A100, A200, Aw/o, and EPI200, the mean values differed significantly over time from baseline to 120 min after injection (*p* < 0.001^(§^*^)^; η_p_^2^ ≥ 0.96). With A100 and A200, it revealed that StO_2_ was significantly different (*p* < 0.001^(§)^) from baseline at all measurement time points except after 30 min (*p* = 0.996^(§)^ and *p* = 0.388^(§)^). With Aw/o, StO_2_ was found to be significantly (*p* < 0.001^(§)^) different from baseline at all measurement time points. With EPI200, StO_2_ was significantly different from baseline at all time points (*p* < 0.01^(§)^) except for 3 min p.i. (0.218^(§)^) (Figure 6).

*Peripheral^ROI−2^*

Overall, for A100, A200, Aw/o, and EPI200, the mean values differed significantly over time from baseline to 120 min after injection (*p* < 0.001^(§^*^)^; η_p_^2^ ≥ 0.93). With A100 and A200, it revealed that StO_2_ was significantly different (*p* < 0.001^(§)^) from baseline at all measurement time points except after 15 min (*p* = 0.057^(§)^ and *p* = 0.851^(§)^). With Aw/o, StO_2_ was found to be significantly (*p* < 0.001^(§)^) different from baseline at all measurement time points. EPI200 revealed that StO2 was significantly different from baseline at all time points (*p* < 0.01^(§)^) except for 3 min p.i. (0.218^(§)^) (Figure 6).

## 4. Discussion

**Figure 7:**
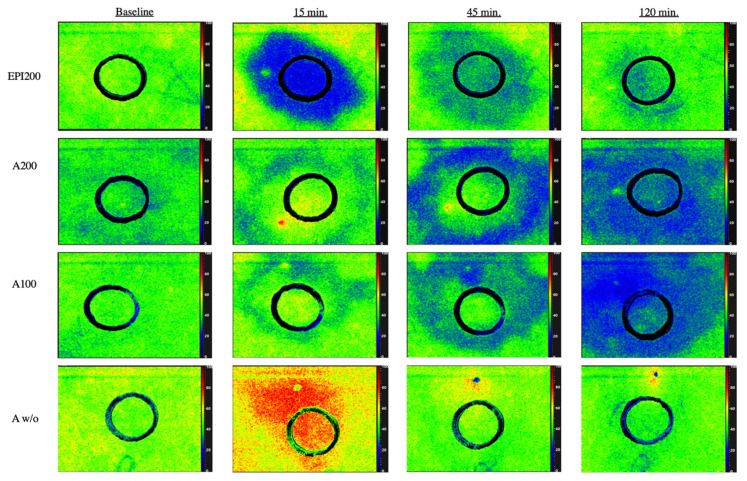
False colour images showing the local dynamic StO_2_ effect per drug group over time from baseline to 15, 45, and 120 min after injection.

This is the first study to investigate the dynamic vasoactive/perfusion effect after the subcutaneous injection of Articain-4% with (A100 and A200) and without (Aw/o) epinephrine in different concentrations as well as pure epinephrine (EPI200) using hyperspectral imaging of the highly sensitive blood flow surrogate parameter StO_2_. As a major result, this study revealed that skin perfusion drops below baseline after 30 min following injection of A100 and A200. Furthermore, there was no difference between A100 and A200. In addition, the duration of vasoconstriction was sustained over a period of >120 min. Finally, a spatial pattern of extending hypoperfusion with epinephrine-containing LA with a primarily marginal circular onset of hypoperfusion with simultaneous hyperaemia around the injection site (centre) was found (Figure 7).

Clinically, the maximum reduction of peripheral blood flow must be set against the risk of local and systemic drug side effects. Nonetheless, appropriate concentrations of epinephrine should be combined with the LA to guarantee sufficient depth and duration of anaesthesia [1,11,21]. Regarding the bloodlessness of the surgical field, there is controversy in the literature about the time interval required between injection and incision. While some authors consider less than 7 min sufficient [22,23], others recommend 13 [5] or even more than 25 min [13]. This could be due to different anatomical regions (eyelid versus forearm, neck, oral mucosa/gingiva), different concentrations of epinephrine or LA, too small case numbers as well as fundamentally different study protocols (e.g., close spacing of the injection sites in the eyelid area with result falsification as well as unsuitable drug combinations compared) and measurement methods (spectroscopy versus blood loss measurement). For example, Hult et al. compared lidocaine-2% with (1:100,000) and without epinephrine in terms of a measurable hypoperfusion based on collected blood volume [22]. Regardless of the fact that subcutaneous injection in the area of the upper eyelid is difficult to standardise in the absence of a regular subcutis and direct muscular support in most cases [24], choosing plain lidocaine-2% as the control site must be rated critically due to vasodilatation by all amide-LA [25,26,27]. This point was taken into account accordingly in the present study by injecting a constant volume of 0.2 mL at a distance of 15 cm between each injection site.

In the present study, epinephrine was used in combination with Articaine-4% at a concentration of 1:100,000 (A100) and +1:200,000 (A200), as well as plain epinephrine 1:200,000 (EPI200). The lack of difference in perfusion reduction between A100 and A200 is consistent with other studies using lidocaine instead of articaine [26,27]. Gessler et al. even found no difference in the ear canal blood flow when comparing lidocaine-1%+epinephrine 1:50,000, 1:100,000, and +1:200,000 [27]. This is in contrast to the results of Moore et al., who demonstrated a greater reduction in blood flow and a clearer surgical field with A100 compared to A200 in the oral setting [3]. For oral LA, no difference in anaesthetic depth was found between A100 and A200 when performing infiltration anaesthesia [21,28]. With regard to the drug effects’ spread, the present study demonstrates for the first time in the literature a ring-shaped ischaemia with the application of A100 and A200 (4). Haemoglobin and the differentiation between its oxygenated and deoxygenated form plays a central role in HSI perfusion analysis. While oxygenated haemoglobin shows a double peak in the wavelength range between 500 and 600 nm and deoxygenated haemoglobin shows a single peak, both differ particularly at 760 nm. Since the absorbance of haemoglobin in the range from 570 to 590 nm is high, electromagnetic radiation of a shorter wavelength shows a lower penetration depth in the tissue; thus, microcirculation (StO_2_) is detected at a depth of up to 1 mm, as it reflects the percentage of haemoglobin oxygen saturation in the capillary area, records arterial and venous blood, and displays changes in oxygen supply and consumption directly on site in the tissue. Uniform standard or limit values do not yet exist, although scientific studies are still underway. The tissue-oxygen-saturation values of healthy volunteers are typically 50%–70% [29]. In the present study, StO_2_ was much higher centrally (ROI-1) than in the peripheral region (ROI-2). This pattern was not seen with plain Aw/o and EPI200. The authors are not aware of any literature that has already described this effect. A plausible cause for this could be a more dynamic spread of epinephrine within the tissue, which is responsible for the marginally observed ischaemia and explains the long-lasting hyperaemic effect around the application site (ROI-1). From our point of view, the frequently described diffusion potential of the local anaesthetic solution towards the nerve (area) must be critically evaluated, which means that, for example, in the case of IANB, the technically correct implementation with application as close to the nerve as possible is of primary importance. Limitations of the study are the lack of association of the measured values to the actual reduction of blood flow after a skin incision as well as the transferability to other body regions such as the head and neck region with a stronger blood supply or the oral mucosa.

## 5. Conclusions

To take advantage of local hypoperfusion for vasoconstriction in the surgical field, it seems advisable to wait at least 30 min after subcutaneous application of Articaine-4% with epinephrine 1:100,000 or 1:200,000 until skin incision. Therefore, the use of epinephrine at or even below 1:100,000 must be discussed in the absence of benefits for anaesthesia and blood flow reduction.

## Data Availability

All raw data on which this study is based will be made available by the corresponding author upon request.

## Supplementary Materials

The following are available online at https://www.mdpi.com/article/10.3390/jcm10153411/s1, Table S1: Shows the means and standard deviations of StO2 for each group, as well as the significance level within ROI-1 and ROI-2, and comparing the two (ROI-1 vs. 2) at Baseline, Table S2: Shows the means and standard deviations of StO2 for each group, as well as the significance level within ROI-1 and ROI-2, and comparing the two (ROI-1 vs. 2) at 30 s. after injection, Table S3: Shows the means and standard deviations of StO2 for each group, as well as the significance level within ROI-1 and ROI-2, and comparing the two (ROI-1 vs. 2) at 1 and 2 min. after injection, Table S4: Shows the means and standard deviations of StO2 for each group, as well as the significance level within ROI-1 and ROI-2, and comparing the two (ROI-1 vs. 2) at 3, 4 and 5 min. after injection, Table S5: Shows the means and standard deviations of StO2 for each group, as well as the significance level within ROI-1 and ROI-2, and comparing the two (ROI-1 vs. 2) at 15 min. after injection, Table S6: Shows the means and standard deviations of StO2 for each group, as well as the significance level within ROI-1 and ROI-2, and comparing the two (ROI-1 vs. 2) at 30 min. after injection, Table S7: Shows the means and standard deviations of StO2 for each group, as well as the significance level within ROI-1 and ROI-2, and comparing the two (ROI-1 vs. 2) at 45 min. after injection, Table S8: Shows the means and standard deviations of StO2 for each group, as well as the significance level within ROI-1 and ROI-2, and comparing the two (ROI-1 vs. 2) at 60 min. after injection, Table S9: Shows the means and standard deviations of StO2 for each group, as well as the significance level within ROI-1 and ROI-2, and comparing the two (ROI-1 vs. 2) at 120 min. after injection.
