# Supplementary material for: Hyperspectral Imaging to Study Dynamic Skin Perfusion after Injection of Articaine-4% with and without Epinephrine—Clinical Implications on Local Vasoconstriction
Source: J Clin Med. 2021 Jul 31;10(15):3411. doi: 10.3390/jcm10153411 (PMC8347280; doi:10.3390/jcm10153411)
Supplement: Supplementary file 1 [file jcm-10-03411-s001.zip › jcm-1259225-supplementary.pdf]

Journal of
Clinical Medicine

MDPI

Table S1. Shows the means and standard deviations of StO_2 for each group, as well as the significance level within ROI-1 and ROI-2, and comparing the two (ROI-1 vs. 2) at Baseline. Significance is considered as $p<0.05$.

					**Mann-Whitney-Test (P-Value)**			
**ROI**	**Time point**	**Formulation**	**Mean (%)**	**SD±**	**A100**	**A200**	Aw/o	**EPI200**
**ROI-1**	**Baseline**	A100	40.45	5.15				
		A200	40.27	6.19	0.82			
		Aw/o	40.11	5.77	0.432	0.279		
		EPI200	41.14	5.54	0.499	0.352	0.874	
**ROI-2**		A100	41.41	4.74				
		A200	40.31	6.71	0.213			
		Aw/o	42.09	4.86	0.192	0.056		
		EPI200	41.52	5.38	0.693	0.246	0.535	
**ROI-1 vs. 2**		A100			0.291			
		A200				0.711		
		Aw/o					0.276	
		EPI200						0.634

Table S2. Shows the means and standard deviations of StO_2 for each group, as well as the significance level within ROI-1 and ROI-2, and comparing the two (ROI-1 vs. 2) at 30 sec. after injection. Significance is considered as $p<0.05$.

					**Mann-Whitney-Test (P-Value)**			
**ROI**	**Time point**	**Formulation**	**Mean (%)**	**SD±**	**A100**	**A200**	**Articaine 4%**	**EPI200**
**ROI-1**	**30 s**	A100	50.30	7.08				
		A200	49.29	6.64	0.754			
		Aw/o	57.76	5.77	<0.001	<0.001		
		EPI200	51.18	7.89	0.446	0.273	<0.001	
**ROI-2**		A100	50,54	6,92				
		A200	48,95	7,76	0.377			
		Aw/o	54,19	5,23	<0.001	<0.001		
		EPI200	57,59	5,83	<0.001	<0.001	<0.011	
**ROI-1 vs. 2**		A100			0.861			
		A200				0.921		
		Aw/o					0.003	
		EPI200						<0.001

Table S3. Shows the means and standard deviations of StO_2 for each group, as well as the significance level within ROI-1 and ROI-2, and comparing the two (ROI-1 vs. 2) at 1 and 2 min. after injection. Significance is considered as $p<0.05$.

					Mann-Whitney-Test (P-Value)			
**ROI**	**Time point**	**Formulation**	**Mean (%)**	**SD±**	**A100**	**A200**	**Aw/o**	**EPI200**
**ROI-1**	**1 min**	A100	48.72	7.59				
		A200	48.31	6.28	0.983			
		Aw/o	58.15	5.73	<0.001	<0.001		
		EPI200	49.71	8.14	0.385	0.292	<0.001	
**ROI-2**		A100	49.05	6.64				
		A200	47.26	7.41	0.268			
		Aw/o	53.59	5.12	<0.001	<0.001		
		EPI200	57.18	6.14	<0.001	<0.001	<0.001	
**ROI-1 vs. 2**		A100			0.726			
		A200				0.482		
		Aw/o					<0.001	
		EPI200						<0.001
**ROI-1**	**2 min**	A100	47.91	8.16				
		A200	49.12	6.66	0.416			
		Aw/o	59.88	5.46	<0.001	<0.001		
		EPI200	44.72	8.61	0.385	0.014	<0.001	
**ROI-2**		A100	47.44	6.75				
		A200	45.77	7.04	0.235			
		Aw/o	54.18	5.10	<0.001	<0.001		
		EPI200	54.97	6.52	<0.001	<0.001	0.828	
**ROI-1 vs. 2**		A100			0.808			
		A200				0.065		
		Aw/o					<0.001	
		EPI200						<0.001

Table S4. Shows the means and standard deviations of StO2 for each group, as well as the significance level within ROI-1 and ROI-2, and comparing the two (ROI-1 vs. 2) at 3, 4 and 5 min. after injection. Significance is considered as $p<0.05$.

					Mann-Whitney-Test (P-Value)			
**ROI**	**Time point**	**Formulation**	**Mean (%)**	**SD±**	**A100**	**A200**	**Aw/o**	**EPI200**
**ROI-1**	**3 min**	A100	48.68	8.25				
		A200	50.44	6.70	0.232			
		Aw/o	62.30	5.50	<0.001	<0.001		
		EPI200	39.42	8.56	<0.001	<0.001	<0.001	
**ROI-2**		A100	46.78	6.588				
		A200	45.79	7.483	0.569			
		Aw/o	55.50	4.951	<0.001	<0.001		
		EPI200	51.59	7.092	<0.001	<0.001	<0.001	
**ROI-1 vs. 2**		A100			0.196			
		A200				<0.001		
		Aw/o					<0.001	
		EPI200						<0.001
**ROI-1**	**4 min**	A100	49.55	8.00				
		A200	51.42	7.09	0.207			
		Aw/o	64.39	5.14	<0.001	<0.001		
		EPI200	35.31	8.51	<0.001	<0.001	<0.001	<0.001
**ROI-2**		A100	46.56	6.61				
		A200	45.99	7.67	0.910			

		Aw/o	56.98	5.15	<0.001	<0.001		
		EPI200	47.61	8.18	0.480	0.398	<0.001	
**ROI-1 vs. 2**		A100			0.029			
		A200				<0.001		
		Aw/o					<0.001	
		EPI200						<0.001
					Mann-Whitney-Test (P-Value)			
**ROI**	**Time point**	**Formulation**	**Mean (%)**	**SD±**	**A100**	**A200**	**Aw/o**	**EPI200**
**ROI-1**	**5 min**	A100	50.08	7.97				
		A200	52.04	7.54	0.190			
		Aw/o	65.88	5.07	<0.001	<0.001		
		EPI200	31.65	8.40	<0.001	<0.001	<0.001	
**ROI-2**		A100	45.65	6.48				
		A200	45.94	7.95	0.426			
		Aw/o	57.97	5.35	<0.001	<0.001		
		EPI200	43.98	8.42	0.154	0.040	<0.001	
**ROI-1 vs. 2**		A100			<0.001			
		A200				<0.001		
		Aw/o					<0.001	
		EPI200						<0.001

Table S5. Shows the means and standard deviations of StO_2 for each group, as well as the significance level within ROI-1 and ROI-2, and comparing the two (ROI-1 vs. 2) at 15 min. after injection. Significance is considered as $p<0.05$.

					**Mann-Whitney-Test (P-Value)**			
**ROI**	**Time point**	**Formulation**	**Mean (%)**	**SD±**	**A100**	**A200**	**Aw/o**	**EPI200**
**ROI-1**	**15 min**	A100	47.14	8.20				
		A200	51.19	8.22	0.011			
		Aw/o	67.31	5.12	<0.001	<0.001		
		EPI200	17.38	7.25	<0.001	<0.001	<0.001	
**ROI-2**		A100	39.76	6.20				
		A200	41.31	8.06	0.041			
		Aw/o	58.60	6.47	<0.001	<0.001		
		EPI200	24.63	6.96	<0.001	<0.001	<0.001	
**ROI-1 vs. 2**		A100			<0.001			
		A200				<0.001		
		Aw/o					<0.001	
		EPI200						<0.001

Table S6. Shows the means and standard deviations of StO_2 for each group, as well as the significance level within ROI-1 and ROI-2, and comparing the two (ROI-1 vs. 2) at 30 min. after injection. Significance is considered as $p<0.05$.

					Mann-Whitney-Test (P-Value)			
**ROI**	**Time point**	**Formulation**	**Mean (%)**	**SD±**	**A100**	**A200**	**Aw/o**	**EPI200**
**ROI-1**	**30 min**	A100	40.59	7.77				
		A200	41.90	8.37	0.258			
		Aw/o	61.17	6.11	<0.001	<0.001		
		EPI200	20.43	6.89	<0.001	<0.001	<0.001	
**ROI-2**		A100	34.545	4.73				
		A200	33.61	6.18	0.238			
		Aw/o	54.71	6.85	<0.001	<0.001		
		EPI200	22.02	6.19	<0.001	<0.001	<0.001	
**ROI-1 vs. 2**		A100			<0.001			
		A200				<0.001		
		Aw/o					<0.001	
		EPI200						0.366

Table S7. Shows the means and standard deviations of StO_2 for each group, as well as the significance level within ROI-1 and ROI-2, and comparing the two (ROI-1 vs. 2) at 45 min. after injection. Significance is considered as $p<0.05$.

					Mann-Whitney-Test (P-Value)			
**ROI**	**Time point**	**Formulation**	**Mean (%)**	**SD±**	**A100**	**A200**	**Aw/o**	**EPI200**
**ROI-1**	**45 min**	A100	33.72	7.09				
		A200	33.95	7.31	0.517			
		Aw/o	54.79	6.58	<0.001	<0.001		
		EPI200	26.39	7.19	<0.001	<0.001	<0.001	
**ROI-2**		A100	30.34	4.34				
		A200	28.80	5.65	0.046			
		Aw/o	50.02	6.63	<0.001	<0.001		
		EPI200	26.73	6.30	<0.001	0.048	<0.001	
**ROI-1 vs. 2**		A100			0.003			
		A200				<0.001		
		Aw/o					<0.001	
		EPI200						0.563

Table S8. Shows the means and standard deviations of StO_2 for each group, as well as the significance level within ROI-1 and ROI-2, and comparing the two (ROI-1 vs. 2) at 60 min. after injection. Significance is considered as p<0.05.

					Mann-Whitney-Test (P-Value)			
**ROI**	**Time point**	**Formulation**	**Mean (%)**	**SD±**	**A100**	**A200**	**Aw/o**	**EPI200**
**ROI-1**	**60 min**	A100	29.96	6.06				
		A200	29.65	5.96	0.907			
		Aw/o	48.16	6.48	<0.001	<0.001		
		EPI200	26.38	6.02	<0.001	0.009	<0.001	
**ROI-2**		A100	29.03	4.25				
		A200	26.79	5.07	<0.001			
		Aw/o	44.83	5.21	<0.001	<0.001		
		EPI200	27.12	5.63	0.013	0.895	<0.001	
**ROI-1 vs. 2**		A100			0.617			
		A200				0.002		
		Aw/o					0.009	
		EPI200						0.549

Table S9. Shows the means and standard deviations of StO_2 for each group, as well as the significance level within ROI-1 and ROI-2, and comparing the two (ROI-1 vs. 2) at 120 min. after injection. Significance is considered as p<0.05.

					Mann-Whitney-Test (P-Value)			
**ROI**	**Time point**	**Formulation**	**Mean (%)**	**SD±**	**A100**	**A200**	**Aw/o**	**EPI200**
**ROI-1**	**120 min**	A100	26.08	3.85				
		A200	23.95	4.37	0.006			
		Aw/o	35.97	5.87	<0.001	<0.001		
		EPI200	25.29	4.87	0.264	0.103	<0.001	
**ROI-2**		A100	28.51	3.52				
		A200	26.26	4.51	<0.001			
		Aw/o	37.73	4.20	<0.001	<0.001		
		EPI200	30.68	5.02	0.005	<0.001	<0.001	
**ROI-1 vs. 2**		A100			<0.001			
		A200				<0.001		
		Aw/o					0.108	
		EPI200						<0.001
